# Enrichment and Broad Representation of Plant Biomass-Degrading Enzymes in the Specialized Hyphal Swellings of *Leucoagaricus gongylophorus*, the Fungal Symbiont of Leaf-Cutter Ants

**DOI:** 10.1371/journal.pone.0134752

**Published:** 2015-08-28

**Authors:** Frank O. Aylward, Lily Khadempour, Daniel M. Tremmel, Bradon R. McDonald, Carrie D. Nicora, Si Wu, Ronald J. Moore, Daniel J. Orton, Matthew E. Monroe, Paul D. Piehowski, Samuel O. Purvine, Richard D. Smith, Mary S. Lipton, Kristin E. Burnum-Johnson, Cameron R. Currie

**Affiliations:** 1 Department of Bacteriology, University of Wisconson-Madison, Madison, Wisconsin, United States of America; 2 Great Lakes Bioenergy Research Center, University of Wisconsin, Madison, Wisconsin, United States of America; 3 Biological Sciences Division, Pacific Northwest National Laboratory, Richland, Washington, United States of America; Smithsonian National Museum of Natural History, UNITED STATES

## Abstract

Leaf-cutter ants are prolific and conspicuous constituents of Neotropical ecosystems that derive energy from specialized fungus gardens they cultivate using prodigious amounts of foliar biomass. The basidiomycetous cultivar of the ants, *Leucoagaricus gongylophorus*, produces specialized hyphal swellings called gongylidia that serve as the primary food source of ant colonies. Gongylidia also contain plant biomass-degrading enzymes that become concentrated in ant digestive tracts and are deposited within fecal droplets onto fresh foliar material as ants incorporate it into the fungus garden. Although the enzymes concentrated by *L*. *gongylophorus* within gongylidia are thought to be critical to the initial degradation of plant biomass, only a few enzymes present in these hyphal swellings have been identified. Here we use proteomic methods to identify proteins present in the gongylidia of three *Atta cephalotes* colonies. Our results demonstrate that a diverse but consistent set of enzymes is present in gongylidia, including numerous plant biomass-degrading enzymes likely involved in the degradation of polysaccharides, plant toxins, and proteins. Overall, gongylidia contained over three quarters of all biomass-degrading enzymes identified in the *L*. *gongylophorus* genome, demonstrating that the majority of the enzymes produced by this fungus for biomass breakdown are ingested by the ants. We also identify a set of 40 of these enzymes enriched in gongylidia compared to whole fungus garden samples, suggesting that certain enzymes may be particularly important in the initial degradation of foliar material. Our work sheds light on the complex interplay between leaf-cutter ants and their fungal symbiont that allows for the host insects to occupy an herbivorous niche by indirectly deriving energy from plant biomass.

## Introduction

Leaf-cutter ants of the genus *Atta* are prevalent consumers of foliar biomass in the New World tropics that play an important role in nutrient cycling and ecosystem engineering in many Neotropical habitats [1–3]. Rather than directly consuming the foliar material they collect, leaf-cutter ants use their forage to cultivate symbiotic gardens that they then consume for food [1,4]. Although the gardens cultivated by leaf-cutter ants contain a variety of microbes [5–10], the primary cultivar of these insects is the basidiomycetous fungus *Leucoagaricus gongylophorus*, which plays a major role in degrading the plant forage of the ants [1,11–15]. In addition to its biodegradative role, this fungus also produces the primary sustenance for leaf-cutter ants in the form of nutrient-rich hyphal swellings called gongylidia [1,16], thereby converting recalcitrant plant polymers into nutrients readily available to its host ants.

A central aspect of this insect-microbe symbiosis is the degradation of plant biomass, and a number of behaviors have evolved in these insects to help them both exclude unwanted microbial pests from their gardens and enhance the biomass degrading capacity of *L*. *gongylophorus* [17–20]. Two examples of these behaviors, termed weeding and fungus grooming, allow the ants to efficiently remove both patches of dead or contaminated fungus garden and selectively filter out spores of foreign fungi, respectively [17]. Moreover, entire fungus gardens are structured into different strata such that fresh foliar material is integrated only in top layers before being degraded in a step-wise process as it moved into lower layers [21,22]. When fresh foliar material is integrated into the top strata, the ants first macerate it into smaller pieces and deposit fecal droplets on top before inoculating fresh cultures of *L*. *gonylophorus* from older strata [1].

Previous work has shown that the fecal droplets of leaf-cutter ants contain plant biomass-degrading enzymes, which we define here to include Carbohydrate Active Enzymes (CAZymes), Fungal Oxidative Lignin-degrading Enzymes (FOLymes), and proteases [23–25]. It has been postulated in previous studies that the deposition of these droplets onto fresh foliar material serves as a form of “pretreatment” [26,27] that initiates the degradation process before *L*. *gongylophorus* is inoculated. Many of the plant biomass-degrading enzymes in ant fecal droplets have been shown to be derived from *L*. *gongylophorus* [28,29], and gene expression analyses have shown that some of these enzymes appear to be highly expressed in gongylidia [30,31], suggesting that the enzymes concentrated by *L*. *gongylophorus* in gongylidia are still active after passing through the digestive tract of the ants. Furthermore, analysis of the *Atta cephalotes* genome has shown a reduction in the total number of encoded proteases compared to other insects [32], suggesting that the loss of these proteases may have been an adaptation allowing *L*. *gongylophorus* enzymes to pass through their digestive systems and become concentrated intact in fecal droplets.

Although the enzymes concentrated by *L*. *gongylophorus* in gongylidia have been shown to have broad hydrolytic activity against a variety of plant polymers [23–25,31,33] and are likely of great import to plant biomass degradation in this insect-fungal symbiosis, the full complement of enzymes present in gongylidia is unknown. Here we used proteomic methods to identify proteins present in the gongylidia of three laboratory-reared *Atta cephalotes* colonies. Our results provide insight into the full spectrum of enzymes used by leaf-cutter ants to pretreat foliar biomass and help clarify the extent of the co-evolutionary adaptations of leaf-cutter ants and their symbiotic fungus that underpin this ecologically-important symbiosis.

## Materials and Methods

### Sample collection

Samples of gongylidia were collected from three laboratory-reared colonies of *Atta cephalotes* fed a diet of maple (*Ace*r) and oak *(Quercus*) leaves three times weekly. No field collections were performed in this study, and because all ant colonies were previously collected no specific permission was required for our experiments. Our analyses involved no endangered or protected species. Fungus garden material was taken from the middle strata of fungus gardens and gongylidia were collected using sterile forceps and a dissecting microscope set at 40X magnification. For each sample approximately 50 mg of gongylidia were placed into 50 uL of sterile water in a 1.5 uL microcentrifuge tube before the mixture was frozen at -80°C prior to protein extraction and proteomic characterization.

### Preparation of gongylidia samples

To lyse cells, frozen gongylidia were placed in an ice cold mortar and pestle and ground under liquid nitrogen for several minutes before being transferred into centrifuge tubes with 0.1 mm zirconia beads and bead beat on a Bullet Blender (Next Advance, Averill Park, NY) for 3 minutes on speed 8. Samples were centrifuged and a methanol/chloroform extraction was performed to separate the protein, metabolites and lipids. Ice cold (-20°C) cholorform:methanol mix (prepared 2:1 (v/v)) was added to the sample in a 5:1 ratio over sample volume and vigorously vortexed. The samples were then placed on ice for 5 minutes and then vortexed for 10 seconds followed by centrifugation at 10,000 x g for 10 minutes at 4°C. The upper water soluble metabolite phase was collected into a glass vial, the lower lipid soluble phase was collected into a separate fresh glass vial, and both samples were dried in a SpeedVac before being stored at -80°C until analysis. The remaining protein interlayer was placed in a fume hood to dry.

The protein pellets were resuspended in 100 mM NH_4_HCO_3_ and assayed with Bicinchoninic acid (BCA) (Thermo Scientific, Rockford, IL) to determine the protein concentration. 2,2,2-Trifluoroethanol (TFE) (Sigma, St. Louis, MO) was added for a final concentration of 50% TFE. The samples were sonicated in an ice-water bath for 1 minute and incubated at 60°C for 2 hours with gentle shaking at 300 rpm. The samples were reduced with 2 mM Dithiothreitol (DTT) (Sigma, St. Louis, MO) with incubation at 37°C for 1 hour with gentle shaking at 300 rpm. Samples were then diluted 5-fold with 50 mM NH_4_HCO_3_, 1 mM CaCl2 for preparation for digestion. Sequencing-grade modified porcine trypsin (Promega, Madison, WI) was added to the sample at a 1:50 (w/w) trypsin-to-protein ratio for 3 hours at 37°C. The samples were concentrated in a SpeedVac (ThermoSavant, Holbrook, NY) to a volume of ~30 μl, acidified with trifluoroacetic acid to pH 4 and then centrifuged at 10,000 x g. The supernatant was cleaned with a strong cation exchange solid phase extraction procedure (SCX-SPE) on an OMIX tip (Agilent technologies, Santa Clara, CA) according to the manufacturer’s instructions. The remaining pellet was re-digested, by resolubilization in 2M thiourea, 7 M urea and 1% CHAPS detergent and incubated at 60°C for 2 hours, reduced with 10mM Dithiothreitol (DTT) with incubation at 37°C for 1 hour, diluted 10-fold with 50 mM NH_4_HCO_3_, 1mM CaCl2. The sample was then re-digested with trypsin for 3 hours at 37°C and cleaned with a strong cation exchange-solid phase extraction procedure (SCX-SPE). The supernatant and pellet samples were combined and concentrated down to ~30 μL using a SpeedVac and a final BCA assay was performed to determine the peptide concentration. The samples were then vialed for mass spectrometric analysis.

### Proteomic Analyses

**Fig 1:**
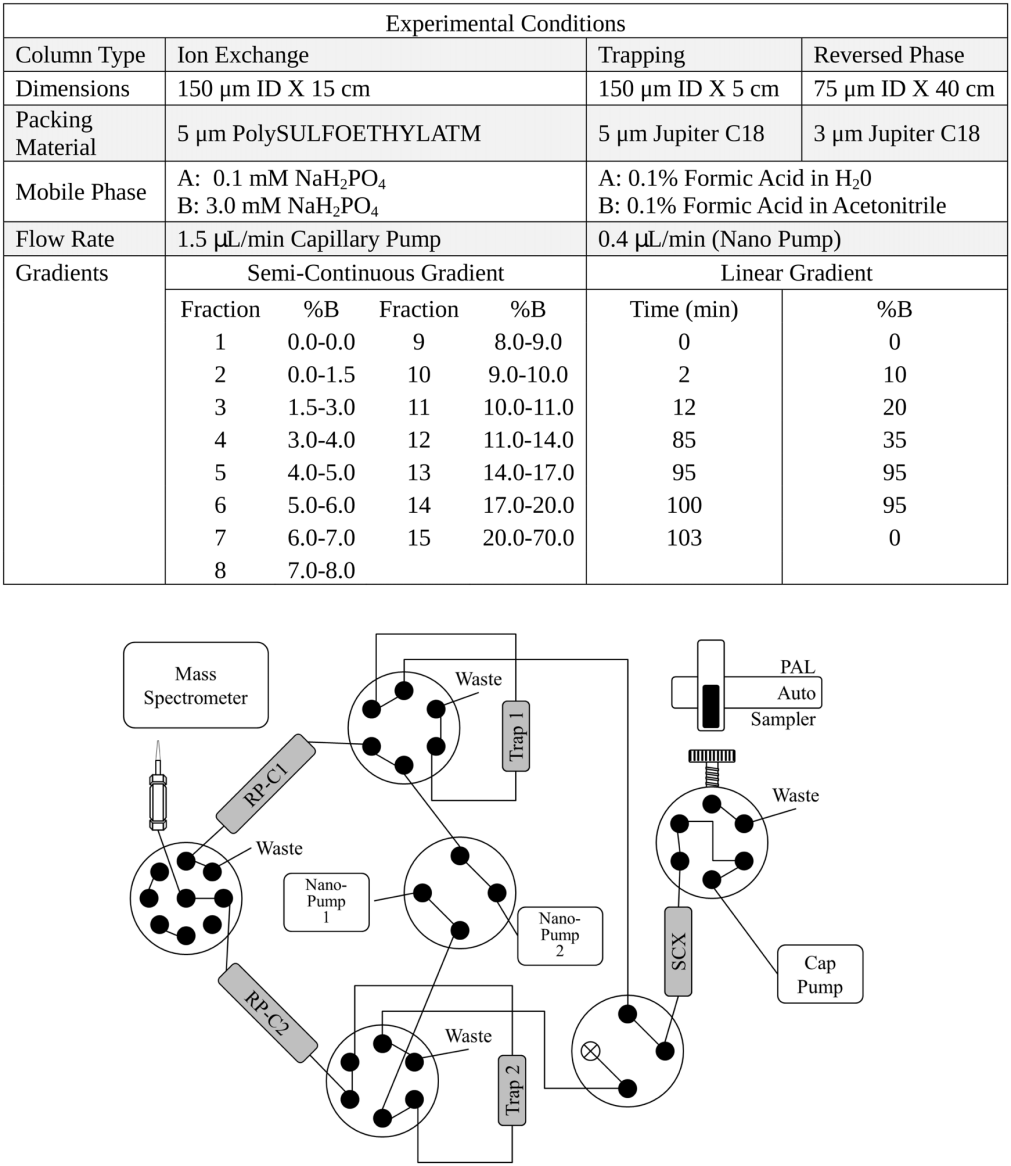
Schematic of the 2D Liquid Chromatography Mass Spectrometry (2D-LC-MS) setup used in this study. SCX: Strong cation exchange.

Due to difficulties in collecting large quantities of gongylidia, on-line multidimensional fractions techniques were utilized in this study to increase measurement coverage given the small sample sizes available. Three biological replicates (5 μg injections) of each sample were analyzed utilizing an on-line 2-D liquid chromatography (LC) separation consisting of one strong cation exchange (SCX) column and two reversed phase C18 columns and two solid phase extraction (SPE) columns for desalting. A total of 15 fractions were collected in this method, and while each fraction was collected, the previously collected fraction was analyzed by the analytical reversed phase separation with detection at the mass spectrometer. This system continuously acquired data for reversed phase separations through all 15 fractions. The 2D-LC system was custom built using two Agilent 1200 nanoflow pumps and one 1200 capillary pump (Agilent Technologies, Santa Clara, CA), various Valco valves (Valco Instruments Co., Houston, TX), and a PAL autosampler (Leap Technologies, Carrboro, NC). Full automation was made possible by custom software that allowed for parallel event coordination and therefore near 100% MS duty cycle through use of two trapping columns and two analytical columns. All columns were manufactured in-house by slurry packing media into fused silica (Polymicro Technologies Inc., Phoenix, AZ) using a 1-cm sol-gel frit for media retention [34]. A detailed schematic of this setup with setting configurations and reagent details is provided in Fig 1.

Mass spectrometry analyses were performed using a Velos Orbitrap mass spectrometer (Thermo Scientific, San Jose, CA) outfitted with a custom electrospray ionization (ESI) interface. Electrospray emitters were custom made by chemically etching 150 um o.d. x 20 um i.d. fused silica [35]. The heated capillary temperature and spray voltage were 275°C and 2.2 kV, respectively. Data were acquired for 100 minutes after a 10 minute delay from when the gradient started. Orbitrap spectra (AGC 1x10^6^) were collected from 400–2000 m/z at a resolution of 100 k followed by data dependent ion trap CID MS/MS (collision energy 35%, isolation width 2.0, AGC 1x10^4^) of the ten most abundant ions. A dynamic exclusion time of 60 sec was used to discriminate against previously analyzed ions using a -0.6 to 1.6 Da mass window.

The peptide tandem mass spectra resulting from these procedures were mapped onto the *L*. *gongylophorus* genome [12] using MS-GF+ [36]. The resulting data were filtered by MS-GF < 1x10^-10^ and mass error < +/- 2.5 ppm which resulted in a spectral level FDR of 1.69% based on a decoy search (S1 Data). For each of the samples, peptide spectral counts were summed across the 15 fractions.

### Enzyme annotations and statistical analyses

Carbohydrate Active Enzymes (CAZymes, [37]), Fungal Oxidative Lignin-degrading enzymes (FOLymes, [38]), and protease annotations (following the MEROPs classification system [39]) for the *L*. *gongylophorus* genome were predicted and annotated to the protein family level, as previously described [12]. Additionally, proteins of interest in the gongylidia samples to which a large number of spectra could be mapped were annotated using homology searches against the Swissprot database [40] (downloaded 5/12/2014) using LAST version 393 [41] (minimum 70% length coverage required, top hits retained). Analysis of the *L*. *gongylophorus* proteins was performed using the gene predictions previously generated using GeneMark-ES [12,42].

Spectral counts for the *L*. *gongylophorus* proteins were compared to those previously reported from proteomic characterization of *Atta cephalote*s whole fungus gardens [12]. Only those spectra previously identified as mapping to *L*. *gongylophorus* proteins were used in this comparison. Fisher’s Exact Test (P < 0.005) was used to determine if the proportion of mass spectra mapping to plant biomass-degrading enzymes or their families was enriched in gongylidia compared to whole fungus gardens (top, middle, and bottom strata combined). For ordination analyses raw spectral counts mapping to proteins identified in the gongylidia and fungus garden samples were normalized using the arcsine square-root transform. Non-metric multidimensional scaling (NMDS) was conducted on these transformed normalized counts using the vegan package in the R statistical programming environment [43,44].

## Results and Discussion

**Fig 2:**
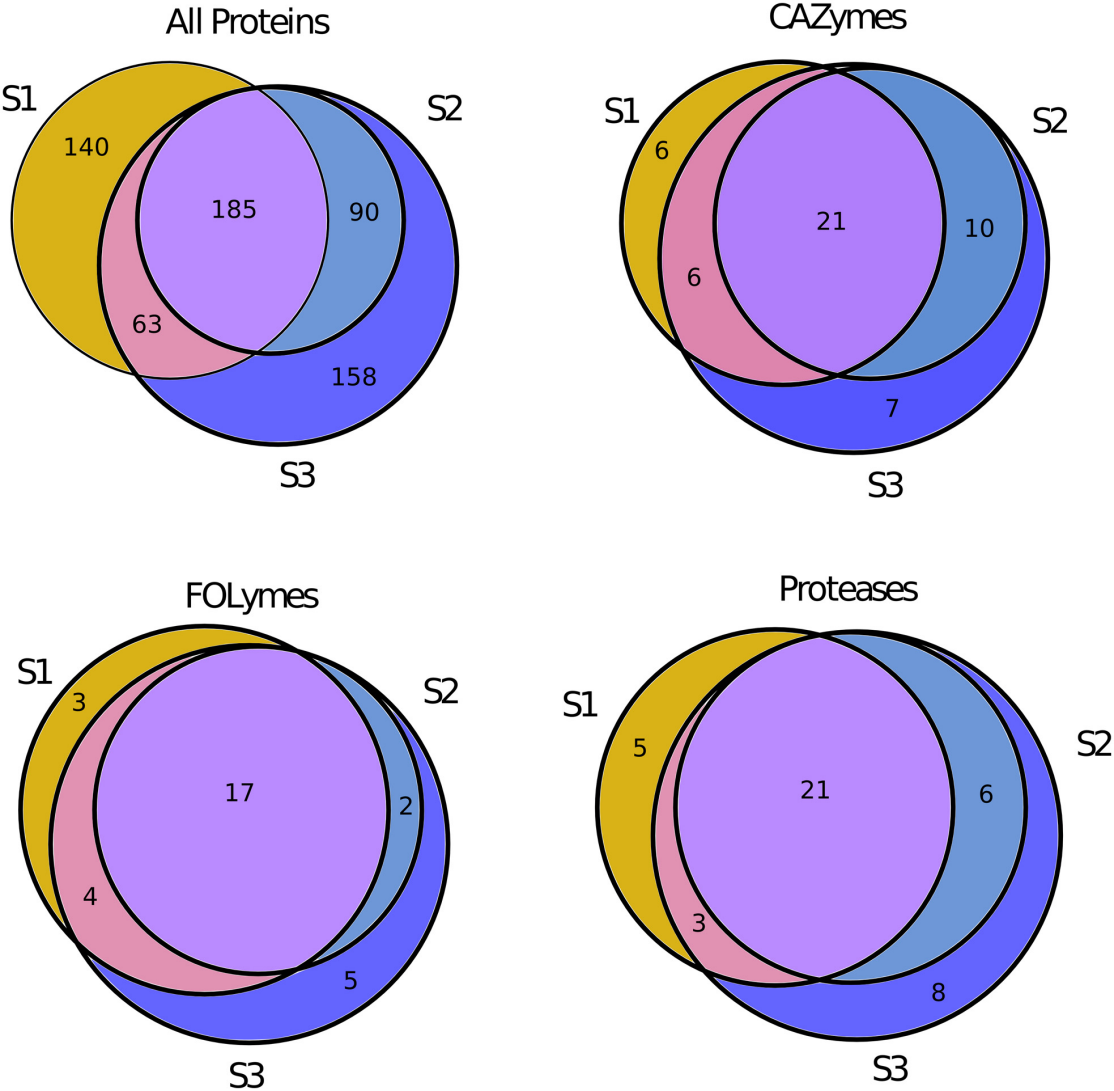
Venn diagram showing shared total proteins, CAZymes, FOLymes, and proteases identified in the three gongylidia samples (labeled S1-S3) analyzed in this study.

We identified a total of 636 *L*. *gongylophorus* proteins using 27,313 total spectral identifications (17,660 unique spectral identifications after quality-filtering [45]) in the three gongylidia samples tested (275–498 proteins identified per sample) (Table 1 and S1 Data). Of these proteins, 123 (19.3%) were predicted plant biomass-degrading enzymes including 50 Carbohydrate Active Enzymes (CAZymes) likely involved in polysaccharide degradation, 31 Fungal Oxidative Lignin-Degrading Enzymes (FOLymes) likely involved in aromatic compound degradation, and 43 proteases likely involved in protein degradation. One of these enzymes (LAG_513) was found to have matches both to family GH5 glycoside hydrolases and family A22B aspartyl proteases. Of the 636 proteins identified, 185 (29%) were found in all three samples, while 338 (53%) were found in at least two samples (Fig 2). Many of the plant biomass-degrading enzymes identified were also shared between samples, with 21 CAZymes (42%), 17 FOLymes (55%), and 21 proteases (49%) identified in all three samples (Fig 2).

**Table 1:** Summary of spectral counts and proteins identified.

Gongylidia Sample	Total Mapped Spectra	Total Proteins Identified	CAZymes Identified	FOLymes Identified	Proteases Identified
Sample 1	2,531	388	32	24	29
Sample 2	9,007	275	30	19	27
Sample 3	15,775	498	37	28	38
Total	27,313	636	49	31	43

**Fig 3:**
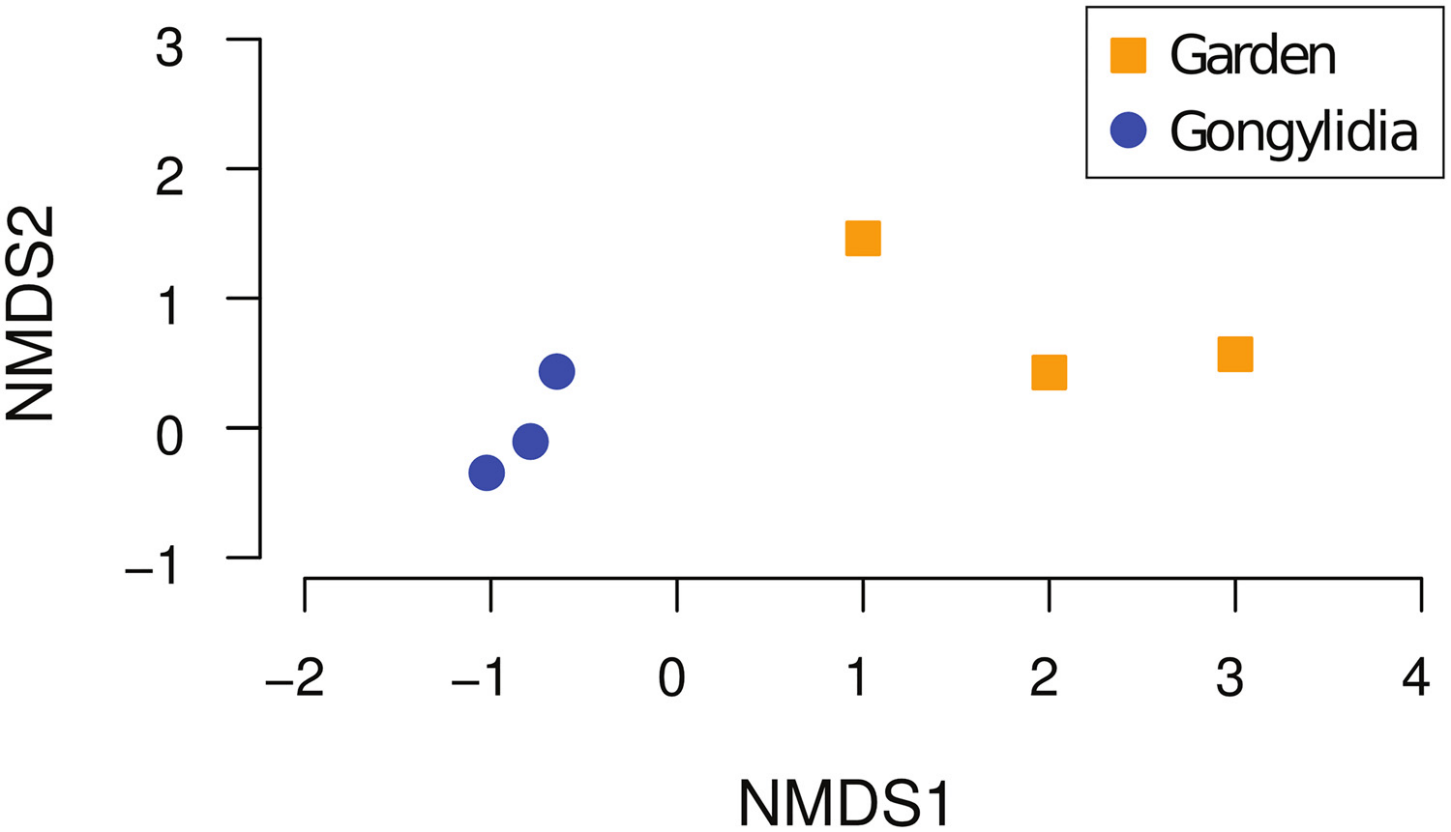
Non-metric multidimensional scaling (NMDS) ordination plot of fungus garden and gongylidia samples. Gongylidia samples are represented by blue circles, while orange squares represent fungus garden samples.

Spectra mapping to CAZymes, FOLymes, and proteases were over-represented in the gongylidia samples compared to spectra mapping to the same enzyme sets in earlier proteomic experiments performed on whole fungus garden material [12] (Fisher’s Exact Test, p < 0.005). Moreover, nonmetric multidimensional scaling (NMDS) analyses of overall protein profiles of the gongylidia and whole fungus garden samples confirmed distinct clustering between these two sample groups (Fig 3). Together, these results indicate that the overall protein content of gongylidia is distinct from that of whole fungus gardens, and that plant biomass-degrading enzymes in particular are enriched in these hyphal swellings. The enrichment of plant biomass-degrading enzymes in gongylidia relative to whole fungus gardens, the large portion of shared proteins in the gongylidia samples (Fig 2), and the overall similarity of gongylidia protein profiles relative to those of whole fungus garden samples (Fig 3) all support the hypothesis that a consistent and specialized set of enzymes is produced in these hyphal swellings.

**Fig 4:**
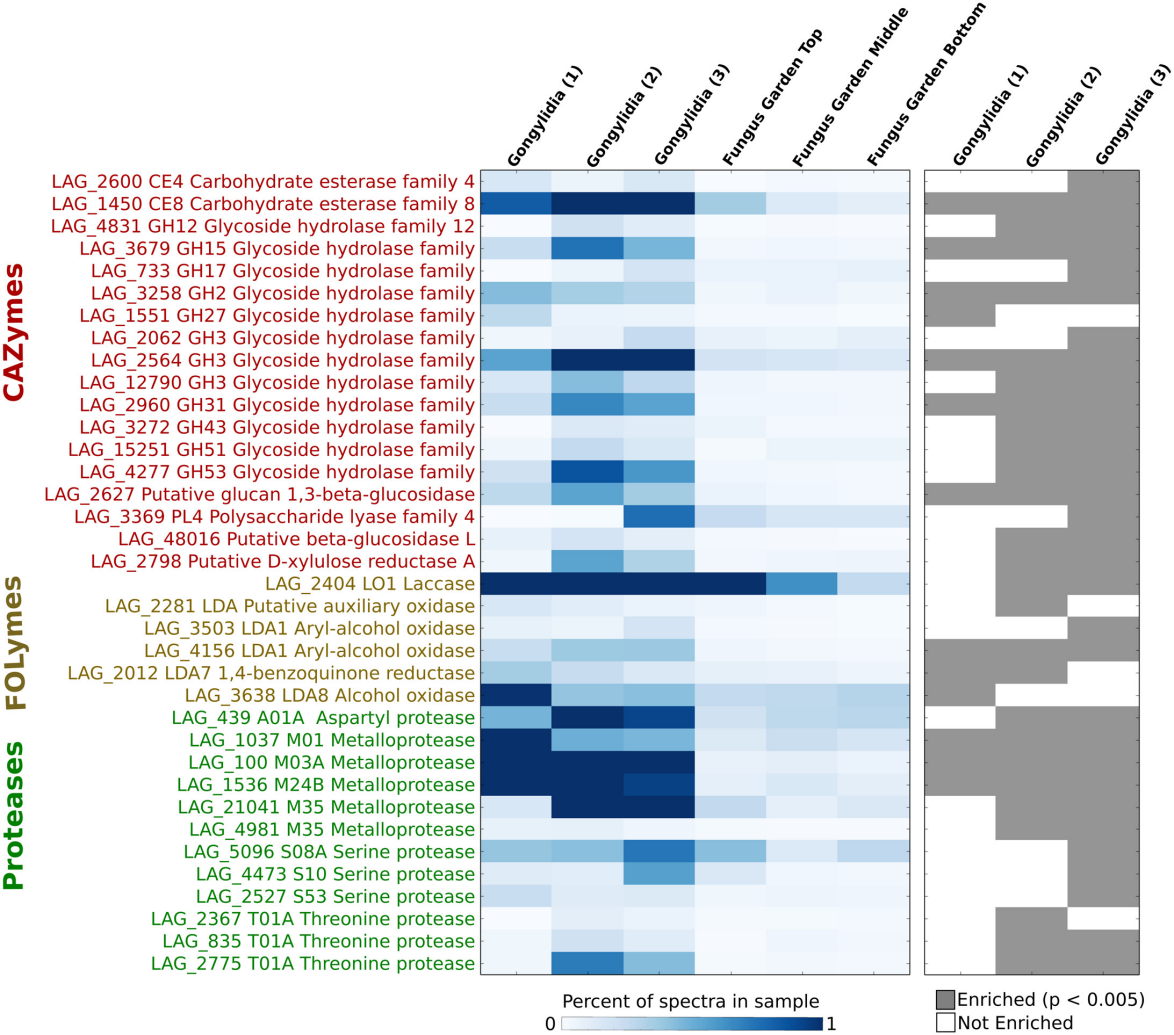
Heatmaps are presented that show the relative percent of total spectra that could be mapped to specific CAZymes, FOLymes, and proteases (left) and those enzymes that were found to be enriched in at least one gongylidia sample (right; Fisher’s Exact Test, p < 0.005). Only enzymes identified as enriched in at least one gongylidia sample compared to all fungus garden samples combined are shown.

Comparisons of the spectral abundance of individual proteins between gongylidia and whole fungus gardens revealed that 129 proteins were enriched in at least one gongylidia sample, and 25 of these proteins were enriched in all three samples (Fisher’s Exact Test, p < 0.005). The enzymes enriched in all three gongylidia samples include 6 CAZymes, 1 FOLyme, and 3 proteases, and another 13 CAZymes, 6 FOLymes, and 11 proteases were enriched in either one or two gongylidia samples (Fig 4). The 6 CAZymes enriched in all three gongylidia samples belong to enzyme families known to catalyze the degradation of pectin (family CE8) and glucosides such as starch (GH2, GH3, GH31). The three proteases enriched in all three gongylidia samples are metalloproteases (LAG_100, LAG_1037, and LAG_1536), consistent with the identification of these enzymes in fecal droplets of *Atta texana* [23,28]. An LO1 laccase enriched in two gongylidia samples (LAG_2404) has previously been implicated in the detoxification of plant defense compounds potentially harmful to *L*. *gongylophorus* or the host ants [31]. The aryl-alcohol oxidase (LAG_4156) enriched in all three samples may play a role similar or auxiliary to laccases, although in other basidiomycetous fungi enzymes of this class have been implicated in hydrogen peroxide generation and lignocellulose degradation [46,47], and so it may serve to target structural polymers in foliar biomass rather than plant defense compounds.

The specialized manner in which leaf-cutter ants cultivate fungi for food has been of great interest to scientists for decades [4], and a central goal of many studies has been to characterize the process through which plant biomass is degraded in fungus gardens and converted into usable nutrients for the host ants [7,12,21,22,48,49]. Many earlier studies of leaf-cutter ants of the genus *Atta* as well as other related fungus-growing ant genera revealed the presence of proteases, glycoside hydrolases, and laccases in the fecal droplets of the ants [23–26,28,33], and many of these studies noted the probable fungal origin of these enzymes. More recently, studies of the leaf-cutter ant *Acromyrmex echinatior* have identified pectinases in the fecal droplets and gongylidia [48], and transcripts encoding for laccases, glycoside hydrolases, and proteases have been identified in the gongylidia of this species [30,31]. Moreover, a great deal of other research has investigated plant biomass degradation in leaf-cutter ant fungus gardens more generally without specific emphasis on gongylidia or fecal droplets [5,7,12,21,27,50–52] Given the important role of gongylidia in supplying key biodegradative enzymes to the ants for subsequent deposition onto plant material, here we have built upon these previous studies by thoroughly identifying the CAZymes, FOLymes, and proteases present in the gongylidia of the leaf-cutter ant *Atta cephalotes* using novel proteomic methods.

Our finding of a broad array of plant biomass-degrading enzymes in the gongylidia of *L*. *gongylophorus* has important methodological implications for assaying the presence and activity of enzymes in leaf-cutter ant fungus gardens. Although a number of recent studies have relied on transcriptomics or mRNA quantification as measures of enzyme occurrence in fungus gardens [30,31,51], the enrichment of CAZymes, FOLymes, and proteases in gongylidia and the likely presence of a broad array of these enzymes in the fecal droplets deposited by the ants on fresh foliar biomass [23–25,28] indicates that transcription and protein presence are decoupled in this environment through the specialized gardening behavior of the leaf-cutter ants. This is likely common in fungus gardens since enzymes can be stored in gongylidia and transported away from where they are produced to sites where they are active. The measurement of mRNA alone is thus likely to provide incomplete and even misleading assessments of the presence of enzymes or their activities in specific locations within fungus gardens. We propose that for the inference of biodegradative processes it is more appropriate to use other techniques that rely on the direct measurement of proteins or their activities, such as the proteomic techniques used here and in other studies [12,48] or the direct measurements of enzymatic activity that have previously been used in fungus gardens [24,49,50].

Our finding that plant biomass-degrading enzymes are enriched in gongylidia supports the hypothesis that these enzymes play an important biodegradative role in leaf-cutter ant fungus gardens. Although only 10 CAZymes, FOLymes, and proteases were enriched in all three gongylidia samples, 36 plant biomass-degrading enzymes were enriched in at least one sample and 123 of these enzymes were identified in total. Recent analysis of the *L*. *gongylophorus* genome identified 145 putative plant biomass-degrading enzymes, and our finding of 118 of these enzymes (81% of the total, excluding the five glycoside hydrolases not reported previously) provides the surprising result that the majority of the biodegradative enzymes produced by the fungal cultivar are ingested by the ants in some quantity. These results strongly implicate the ingestion of plant biomass-degrading enzymes and subsequent deposition on fresh foliar material within fecal droplets as a critical aspect of the degradation of plant biomass in fungus gardens. The initial pretreatment of biomass with a diverse set of enzymes prior to inoculation may release free sugars or more labile substrates critical for the initial establishment and sustained growth of *L*. *gongylophorus* in fungus gardens. The apparent lack of cellulases in gongylidia indicates that these enzymes are not part of the pretreatment step, possibly because cellulose is used later in the degradation process. These results may partly explain why *L*. *gongylophorus* has been shown to grow slowly or not at all on numerous plant polymers in pure culture despite its apparent rapid growth in fungus gardens, where presumably the specialized gardening behaviors of the ants enhance biomass breakdown [1,11,14,53]. Together with these earlier results, our finding of widespread plant biomass-degrading enzymes in gongylidia therefore underscores the importance of the specialized gardening behaviors of leaf-cutter ants to biomass degradation as well as the extent to which leaf-cutter ants have co-evolved with their symbiotic fungus to efficiently derive energy from foliar material.

## Supporting Information

S1 Data Details regarding spectral identifications, the total number of spectra mapping to proteins in the samples, and enzyme annotations. (XLSX)
