# Correction: Enrichment and Broad Representation of Plant Biomass-Degrading Enzymes in the Specialized Hyphal Swellings of *Leucoagaricus gongylophorus*, the Fungal Symbiont of Leaf-Cutter Ants

**DOI:** 10.1371/journal.pone.0139151

**Published:** 2015-09-23

**Authors:** Frank O. Aylward, Lily Khadempour, Daniel M. Tremmel, Bradon R. McDonald, Carrie D. Nicora, Si Wu, Ronald J. Moore, Daniel J. Orton, Matthew E. Monroe, Paul D. Piehowski, Samuel O. Purvine, Richard D. Smith, Mary S. Lipton, Kristin E. Burnum-Johnson, Cameron R. Currie

An earlier version of Fig 4 was published. Please view the correct Fig 4 here.

**Fig 4:**
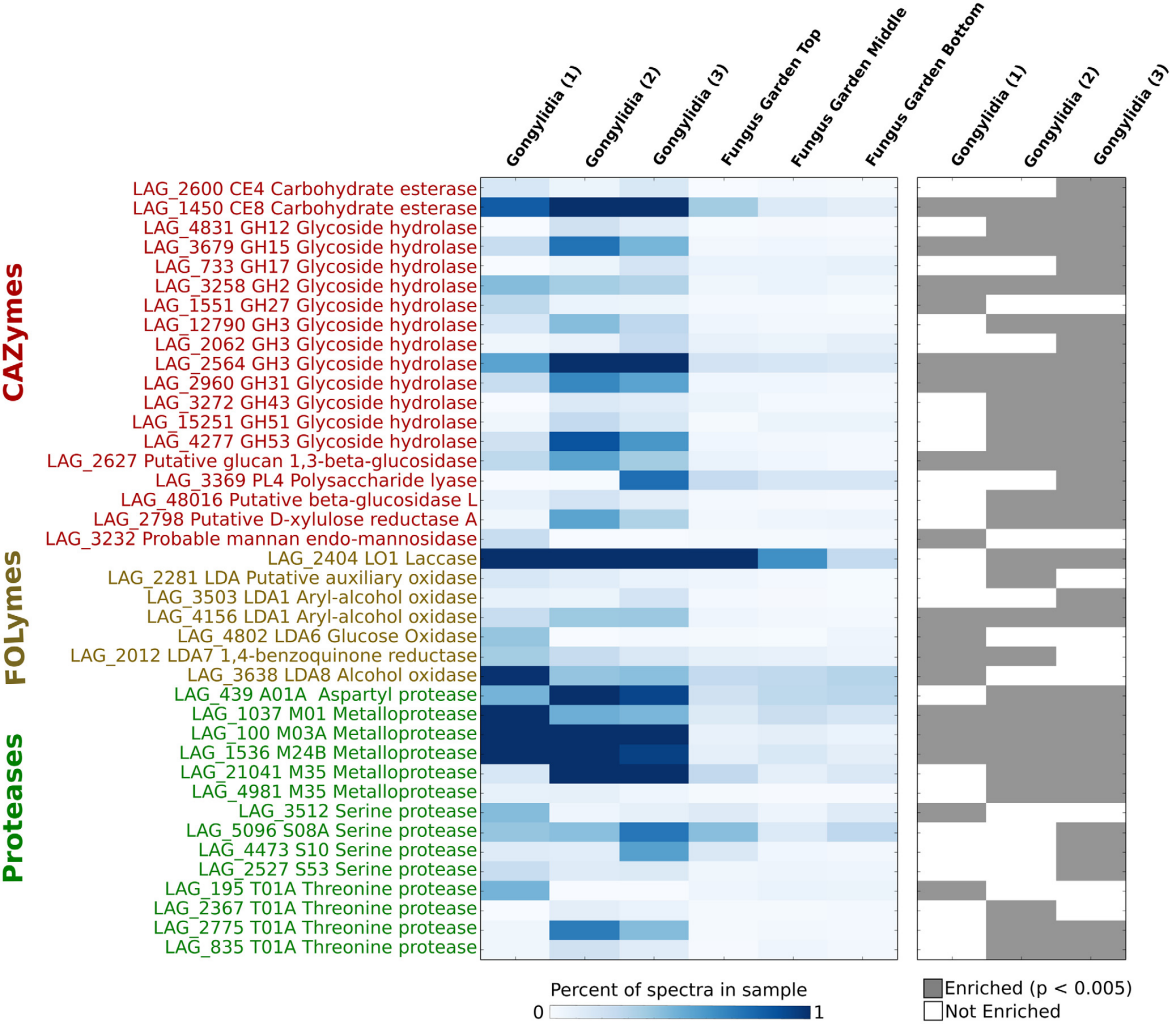
Heatmaps are presented that show the relative percent of total spectra that could be mapped to specific CAZymes, FOLymes, and proteases (left) and those enzymes that were found to be enriched in at least one gongylidia sample (right; Fisher’s Exact Test, p < 0.005). Only enzymes identified as enriched in at least one gongylidia sample compared to all fungus garden samples combined are shown.

There is an error in the second sentence of the final paragraph of the Results and Discussion section. The correct sentence is: Although only 10 CAZymes, FOLymes, and proteases were enriched in all three gongylidia samples, 40 plant biomass-degrading enzymes were enriched in at least one sample and 123 of these enzymes were identified in total.
